# Health Pregnancy, Healthy Baby: testing the added benefits of pregnancy ultrasound scan for child development in a randomised control trial

**DOI:** 10.1186/s13063-019-3924-0

**Published:** 2020-01-06

**Authors:** Linda Richter, Wiedaad Slemming, Shane A. Norris, Alan Stein, Lucilla Poston, Dharmintra Pasupathy

**Affiliations:** 1 DST-NRF Centre of Excellence in Human Development, University of the Witwatersrand, Johannesburg, South Africa; 2 Department of Paediatrics, University of the Witwatersrand, Johannesburg, South Africa; 3 SAMRC Developmental Pathways to Health Research Unit, University of the Witwatersrand, Johannesburg, South Africa; 4 Department of Psychiatry, University of Oxford, Oxford, UK; 5 MRC/Wits Rural Public Health and Health Transitions Research Unit (Agincourt), University of the Witwatersrand, Johannesburg, South Africa; 6 Department of Women & Children’s Health, King’s College London, London, UK

**Keywords:** Fetal ultrasound scan, Antenatal attachment, Early childhood development, Mother–child interaction, Growth, Pregnancy/antenatal

## Abstract

**Background:**

The 2016 World Health Organization Antenatal Guidelines and the 2015 South African Maternal and Child Health Guidelines recommend one early antenatal ultrasound scan to establish gestational age and to detect multiple pregnancies and fetal abnormalities. Prior research indicates that ultrasound scan can also increase parental–fetal attachment. We aim to establish whether, compared to routine care, messages to promote parental attachment and healthy child development, conducted during one or two pregnancy ultrasound scans, improve early child development and growth, exclusive breastfeeding, parental–child interactions and prenatal and postnatal clinic attendance.

**Methods:**

The effect of messages to sensitise mothers and fathers to fetal development will be tested in a three-armed randomised trial with 100 mothers and their partners from Soweto, Johannesburg in each arm. The primary outcome is child development at 6 months postnatally. Secondary outcomes include infant feeding, parental attachment and interaction, parental mental health and infant growth, assessed at 6 weeks and 6 months. Parents in Arm 1 receive a fetal ultrasound scan < 25 weeks during routine antenatal care at tertiary hospitals, and a second standard ultrasound scan at the research site within 2 weeks. Arm 2 participants receive the routine antenatal ultrasound scan and an additional ultrasound scan < 25 weeks at the research site, together with messages to promote parental attachment and healthy child development. Arm 3 participants receive the routine ultrasound scan and two additional ultrasound scans at the research site, < 25 weeks and < 36 weeks, together with messages to promote parental attachment and healthy child development.

**Discussion:**

Evidence from high-income countries suggests that first-time prospective mothers and fathers enjoy seeing their fetus during ultrasound scan and that it is an emotional experience. A number of studies have found that ultrasound scan increases maternal attachment during pregnancy, a predictor of positive parent–infant interactions which, in turn, promotes healthy infant development. It is generally agreed that studies are needed which follow up parental–child behaviour and healthy child development postnatally, include fathers and examine the construct in a wider diversity of settings, especially in low and middle-income countries. Testing the added benefits of pregnancy ultrasound scan for child development is a gap that the proposed trial in South Africa seeks to address.

**Trial registration:**

Pan African Clinical Trials Registry, PACTR201808107241133. Registered on 15 August 2018.

## Background

More than 250 million children younger than 5 years of age in low and middle-income countries (43% of the total globally) are at risk of not reaching their developmental potential because they are stunted or live in extreme poverty. This rises to 70% of young children in sub-Saharan Africa, which has the highest risk burden in the world [1]. In South Africa (SA), the risk is 39% of children under 5 years of age, with knock-on losses of human capital due to inadequate school learning and low earning capacity [2]. The Global Strategy for Women’s, Children’s and Adolescents’ Health [3] and the fourth Sustainable Development Goal [4] recognise the importance of promoting early childhood development (ECD) to ensure that children survive *and* thrive, and rightfully fulfil their human potential and contribute productively to their families and societies.

The 2017 *Lancet* Series *Advancing Early Childhood Development: From Science to Scale* recommended that the contacts which the Reproductive, Maternal, Newborn and Child Health (RMNCH) services have with pregnant women, parents and families are important entry points to promote ECD [5]. Further, the series noted that many effective programmes for ECD are currently built on health services, demonstrating the feasibility of this approach for taking interventions to promote ECD to scale [2, 6].

In 2015, South Africa adopted a National Integrated Early Childhood Development Policy that mandates the health sector to lead in providing services for children and families in the first 1000 days of life, starting from conception, in collaboration with other relevant sectors. Taking this up, the National Department of Health (NDoH) launched a Side-by-Side campaign [7] to bolster support for pregnant women and families with young children, and revised the Road to Health Booklet to emphasise survive and thrive.

The 2015 NDoH Guidelines for Maternity Care in SA [8] recommend that all pregnant women attending a district hospital should have access to one ultrasound scan (US) at 18–20 weeks gestation. The purpose of the US is to confirm an intra-uterine pregnancy, fetal viability, the number of fetuses, gestational age, location of the placenta and amniotic fluid volume. The 2016 WHO Antenatal Guidelines recommend one US before 24 weeks gestation “to estimate gestational age, improve detection of fetal abnormalities and multiple pregnancies, reduce induction for post-term pregnancy, and improve a women’s pregnancy experience” [9], Recommendation B.2.4).

Taking advantage of the SA and WHO recommendations that pregnant women have an ultrasound examination before 24 weeks gestation, the aim of this three-arm trial is to assess whether messages to promote ECD delivered during pregnancy and one or two additional ultrasound examinations will increase parental investment in ECD, as indexed by infant development, nutrition and growth, and the quality of parent–child relationships 6 weeks and 6 months post partum.

### Benefits of pregnancy ultrasound scan

In addition to the survival and health benefits of US reviewed by the WHO in preparation of the guidelines [10], the possibility is raised that US may increase antenatal clinic (ANC) use [11], particularly in view of the recommendation that ANC visits be increased from four to eight. However, the evidence on ultrasound examination, including any psychological benefits, is derived almost solely from high-income countries (HICs).

Early pregnancy US has been routine in HICs for more than 30 years. At the time of introduction of US, studies reported positive parental reactions to seeing images of the fetus [12, 13], that parents enjoyed the experience and that they found it useful to receive information during US [14]. Reading et al. [15] commented on the potential of US to change parental behaviour for the better, such as smoking cessation during pregnancy. Rothenberg speculated that further research could “lead to the development of ultrasound use patterns designed to enhance maternal attachment”, and that “the psychosocial dimension of ultrasound scanning seems obvious to patients and personnel in the field” [16], p. 171).

In qualitative studies, both mothers and fathers report enjoying the US experience and that the presence of the father seemed to have a beneficial effect on the mother [17]. A 1999 review concluded that there was as yet no evidence of increases in health behaviours following US, nor that “seeing the baby” stimulates parental–fetal attachment [18]. Based on their review, Baillie et al. cautioned that “Practitioners and decision makers should be wary of over-interpreting enjoyment of a procedure as evidence of its therapeutic power” ([18], p. 149). Later studies, however, report positive effects of US on maternal and paternal attachment [19–22] but only a few have examined the effects of US on attachment after birth [23, 24].

US studies conducted in sub-Saharan Africa have principally focused on US costs, maintenance, training, human resources, acceptability and other aspects of feasibility, including in Ghana [25], Kenya [26], Zambia [27], Nigeria [28–30], Rwanda [31], South Africa [32], Tanzania [33, 34] and Uganda [35, 36]. In general, US is thought to enhance antenatal care through confirmation of pregnancy status and more accurate estimation of gestational age, but concerns remain about feasibility of wide-scale use in low and middle-income countries and the potential misuse of US for sex determination in countries with a high preference for male children [37]. A recent, large, cluster-randomised control trial in Pakistan, Kenya, Zambia, DRC and Guatemala suggested that US did not increase ANC use or hospital births [38]. However, a review in sub-Saharan Africa concluded that “The potential benefits of obstetric ultrasound are yet to be fully realized in SSA due to challenges ranging from individual patients to institutional and national policies. While there may be conflicting evidence on the impact of universal access on perinatal outcomes, the region presents many research opportunities that could provide answers to these questions” [39], p. E58).

Point-of-care ultrasound (PoCUS) makes scale-up more feasible [39, 40] and evidence for its use more positive than standard US. A recent review [41] concluded that in every study in which it was examined in low and middle-income countries, PoCUS increased antenatal clinic attendance and assisted with the identification and treatment of conditions associated with maternal and neonatal morbidity in under-resourced settings. PoCUS thus has the potential to bring much-needed services to women in rural communities and in poor countries, many of whom need these services most. In addition to increasing access to services, US has also been found to provide an opportunity to engage fathers [42], with the potential of garnering support for the expectant mother and their child and potential sharing of child-care responsibilities.

This study examines US augmented with messages about fetal and infant development and the importance of parent well-being and infant care on postnatal care and early childhood development. We hypothesise that one or two USs with messages about fetal and infant development will have a positive effect on infant development 6 months postnatally, as well as on parental well-being, parent–child attachment, infant feeding and growth and use of preventive health-care services.

## Methods

### Objectives

The trial will evaluate the potential benefits of US on early childhood development and child care by augmenting a routine antenatal service currently provided mainly in tertiary public health facilities in South Africa, with plans to be rolled out to district hospitals. The goal of the intervention is to increase parental investment not only in child survival, but also for the well-being and healthy growth and development of their child. We aim to establish whether, compared to routine care, messages about fetal and child development delivered during one (< 25 weeks) or two (< 25 weeks and again < 36 weeks) fetal USs improve ECD and growth, exclusive breastfeeding, mother/father–infant attachment, maternal and paternal well-being, father involvement and prenatal and postnatal clinic attendance.

Specific objectives are to evaluate whether child development messages delivered during one or two USs:

- Improve early child development
- Improve infant breastfeeding practices and healthy child growth
- Improve parent–infant attachment and interaction during pregnancy and 6 months after birth
- Increase paternal engagement in a young child’s development and care

### Design

Eligible mothers will be randomised to one of three arms: Arm 1, the control group, will receive standard practice of care during pregnancy; Arm 2 will receive the child development intervention at the first research ultrasound visit; and Arm 3 will receive the intervention at two research ultrasound visits. All mothers, infants and partners (where available) will be followed-up 6 weeks and 6 months postnatally for assessments. Participants will continue to receive routine antenatal, perinatal and postnatal care as provided by the tertiary care facility from which all participants are being recruited.

### Setting

The study is situated in Soweto, South Africa. Soweto is adjacent to Johannesburg and is the most populous Black urban residential area in South Africa, with over 1.3 million people counted in the most recent census [43]. Participants will be recruited from women attending antenatal care at Chris Hani-Baragwanath Academic Hospital (CHBH), a tertiary teaching hospital that attends to over 22,000 births annually. Women are referred to CHBH from community health centres with risk factors including HIV and hypertension. Recruited women will attend their study ultrasound visits at the SAMRC Development Pathways for Health Research Unit (DPHRU), which is located on the grounds of CHBH and in walking distance of the CHBH antenatal services.

### Procedures

#### Recruitment

Approximately 500 women present to CHBH each month for their first pregnancy US, of which 50% are < 25 weeks gestation. This offers a potential pool of 250 women per month to be included in the study. Participants will be recruited from women attending the Foetal Medicine Unit (FMU) at CHBH, where routine fetal ultrasound examinations are conducted. Eligible women will be resident within Soweto, aged 18 years and older, with a singleton pregnancy and presenting < 25 weeks gestation. Major fetal abnormalities detected during the first routine ultrasound examination conducted by an experienced radiologist in the FMU, or severe maternal morbidities, will exclude a pregnant woman from the trial. These women will be transferred to specialised clinical services at CHBH, where she will receive appropriate treatment.

Sample size calculations were based on detecting a 0.5-standard deviation (SD) difference between arms on both the Bayley III Language and Cognitive Scales, and *z*-scores for weight-for-age (WAZ), length-for-age (LAZ) and weight-for-length (WLZ) measured at 6 months of age at 90% power, which is 85 per arm. Allowing for 15% potential attrition during pregnancy and during follow-up to 6 months of infant age, the sample size in each of the three arms will be 100. This makes a total sample size of 300 women for recruitment.

### Intervention

Women will be randomised to one of three arms on recruitment at CHBH < 25 weeks gestation after having a fetal ultrasound examination as part of routine antenatal hospital services. The women will be blind to their arm allocation and remain blinded until study completion. Using computer-generated simple randomisation, each consecutive woman screened will be randomly allocated to a study arm. Research assistants responsible for screening will receive the study arm numbers for allocation from the study coordinator on a daily basis. The allocation sequence will be concealed from the research assistants enrolling the participants. Women recruited for Arm 1 will act as the control group and will receive one further standard US at DPHRU < 25 weeks gestation, when they will receive standard fetal growth measurements but no child development messages.

In addition to their routine antenatal US at CHBH, women recruited to Arm 2 will receive an additional US < 25 weeks gestation at DPHRU, during which they will receive messages to promote early childhood development. During the US session, the sonographer will talk with the mother and partner, if present, about the fetus’ sensory development and capacity for learning, fetal responsivity to the environment, maternal well-being and the dependence of the fetus on maternal and paternal health and well-being before and after birth.

In addition to their routine antenatal US at CHBH, women recruited to Arm 3 will receive two additional USs at DPHRU, one < 25 weeks gestation (early US) and another < 36 weeks gestation (late US). They will receive the same child development messages as mothers in Arm 2 at the first US. During the second US, in addition to childhood development messages, the sonographer will encourage the woman and her partner, if present, to personalise the baby, strengthen intentions to exclusively breastfeed, talk and sing to the child before and after birth, and counsel them on how to respond to their young infant’s emotional and learning needs.

Partner involvement and interest in the baby is encouraged by inviting partners to attend the ultrasound sessions of women in all three arms. For mothers recruited to Arms 2 and 3, digital ultrasound images and a photograph of the woman (with her partner where present) having the US will be sent to parents via WhatsApp and printed images given to the parents. A small baby book with the child development messages, as appropriate to the arm, is given to the mothers in Arms 2 and 3, and they are encouraged to paste the printed pictures into the book and to share the US images and photographs with family and friends.

Two qualified and registered sonographers will be trained to deliver the intervention and a Philips HD9 Ultrasound System will be used to conduct all USs. Due to the nature of the intervention, the sonographers will not be blind to the mother’s arm allocation.

### Data collection

Study data will be collected and managed using REDCap (Research Electronic Data Capture) hosted by the University of the Witwatersrand. Data will be collected on the enrolled women, child and partners (where appropriate) at three stages: stage one, baseline at first research ultrasound visit at DPHRU < 25 weeks; stage two, 6-week postnatal follow-up; and stage three, 6-month postnatal follow-up. Data will be collected on tablets at stages one and two by two trained data collectors under the supervision of the study coordinator. At stage three, data will be collected by occupational therapists or physiotherapists trained in administering child development measures, under the supervision of the study coordinator. All data collectors will be trained on the principles of research ethics, Good Clinical Practice, referral procedures and management of difficult situations in the field.

Table 1 presents the schedule of enrolment, interventions and data collection.

**Table 1:** Standard Protocol Items: Recommendations for Interventional Trials (SPIRIT) schedule of enrolment, interventions and data collection

	Study period				
	Enrolment	Intervention		Follow-up	
Timepoint	< 22 weeks GA	< 25 weeks GA	< 36 weeks GA	6 weeks postnatally	6 months postnatally
Enrolment					
Eligibility screen	X				
Informed consent	X				
Random allocation	X				
Interventions					
Early US		X			
Early US with messages		X			
Early and late US with messages		X	X		
Assessments: baseline and antenatal					
Socio-demographic questionnaire	X				
Fetal growth assessment		X	X		
Social support		X	X		
Maternal and paternal antenatal depression and anxiety		X	X		
Maternal and paternal antenatal attachment		X	X		
Maternal and paternal antenatal depression		X	X		
US experiences questionnaire		X	X		
Outcomes: primary and secondary					
Bayley Scales of Infant and Toddler Development, Third Edition					X
Home Screening Questionnaire					X
Mother/father–infant interaction					X
Perinatal and postnatal information				X	
Infant behaviour (crying, sleeping, etc.)				X	
Breastfeeding practices questionnaire				X	X
Maternal and paternal postnatal depression and anxiety				X	X
Social support				X	X
Partner involvement				X	X
Infant growth – weight and length				X	X
Immunisation status				X	X
Clinic attendance				X	X

*GA* gestational age, *US* ultrasound scan

The data collected at each stage is as follows:

- *Baseline at first research ultrasound scan at DPHRU.* Data at the first research visit (< 25 weeks) will include the participant’s contact details, demographic characteristics and reproductive history, socio-economic status, social and instrumental support, and breastfeeding intentions. Maternal and paternal fetal attachment is assessed using the Maternal and Paternal Attachment Scales [44, 45], and the mother and her partner, if available, will complete a social support questionnaire and the Edinburgh Postnatal Depression Scale [46]. The woman and her partner, if available, will also be asked to complete an ultrasound experience questionnaire after the US.

- *Six-week postnatal follow-up.* Two trained outcome assessors, blind to the mother’s arm allocation, will complete the follow-up questionnaires with the mothers, infants and partners, where available. Questionnaires will assess perinatal and postnatal information, breastfeeding practices, and partner and social support. Postnatal depression will be assessed for the mother and partner using the Edinburgh Postnatal Depression Scale. Infant growth will be assessed using standard procedures for infant weight and length (the SECA 416 infantometer for length and the SECA 367 infant scale for weight). Measures will be converted to *z*-scores using the 2006 WHO Growth Standards for LAZ, WAZ and WLZ. Birth weight and length as well as key information on maternal and infant birth outcomes will be collected from the infant’s Road to Health Booklet.

- *Six-month postnatal follow-up.* Occupational therapists or physiotherapists trained in child development will complete the 6-month follow-up assessments with the mothers, infants and partners, where available. The primary outcome for the 6-month follow-up is child development as measured by the Bayley Scales of Infant and Toddler Development, Third Edition [47].

All of the study outcome measures are presented in Table 2.

**Table 2:** Study outcome measures

Outcome	Instrument	Measure	Description	Method of aggregation	Timepoint
Primary outcome					
Child development at 6 months	Bayley Scales of Infant and Toddler Development, Third edition (Bayley III)	The total score from each developmental domain	Bayley III are an individually administered assessment of a child’s attainment of developmental milestones across five domains (i.e. cognitive, language, motor, social–emotional and adaptive skills) [47]. They have been recognised internationally as the most comprehensive tool to assess children from as young as 1 month old and have been validated in South Africa [48]	Individual scores to be compared between intervention and control groups using linear or logistic regression models dependent on the distribution of the data	End point
	Home Screening Questionnaire (HSQ)	HSQ score	The HSQ is a 30-item parent-report tool used to identify features of the home environments related to childhood development [49]. The HSQ has been validated and used for research purposes in a number of countries, including South Africa [50]	Individual scores to be compared between intervention and control groups using linear or logistic regression models dependent on the distribution of the data	End point
Secondary outcomes					
Mother/father–infant interaction at 6 months	Mother/father interaction rating scale	Interaction score	Rated from a 5-min videotape of mother/father and infant “talking and playing with each other”, with the infant in a high chair, the mother sitting on a chair at the same height opposite the infant and an attractive stack toy for them to share. We will use an adapted coding scheme developed by Richter et al. to code engagement (eye contact, joint attention), emotional tone, emotional regulation (maternal/paternal comforting for distress), maternal/paternal responsiveness and maternal/paternal scaffolding [51]	Individual scores to be compared between intervention and control groups using linear or logistic regression models dependent on the distribution of the data	End point
Infant growth at 6 weeks and 6 months	Weight and length	Weight (g) Length (cm) Weight-for-age *z*-score (WAZ) Length-for-age *z*-score (LAZ) Weight-for-length *z*-score (WLZ)	Infant growth will be measured using standard procedures for infant weight and length, and converted to *z*-scores using the 2006 WHO Growth Standards (WAZ, LAZ and WLZ)	Differences in mean weight and length of infants will be compared between intervention and control groups	Intermediate and end point
Breastfeeding practices at 6 weeks and 6 months	Maternal self-report questionnaire	Breastfeeding initiation and duration	Information on breastfeeding practices will be collected by trained interviewers using a semi-structured questionnaire	Categorical data will be presented as numbers and percentages. Changes in breastfeeding practices will be compared between intervention and control groups	Intermediate and end point
Maternal and paternal postnatal depression and anxiety during pregnancy, at 6 weeks and at 6 months	Edinburgh Postnatal Depression Scale (EPDS)	EPDS score	The EPDS is a screening tool for antenatal and postnatal depressive symptoms among women. The EPDS has been validated for use antenatally and postnatally in a number of settings, including South Africa [52, 53]	Proportion of mothers/partners showing signs indicative of depression will be compared during pregnancy, at 6 weeks and at 6 months. Data will be summarised by mean, SD and range at each time point	Baseline, intermediate and end point
Immunisation status and clinic attendance at 6 weeks and 6 months	Road to Health Booklet (child health record) or maternal/paternal self-report	Clinic attendance at routine child services (immunisation, growth monitoring, developmental screening, vitamin A, deworming). Other health contacts (e.g. sick child care)		Categorical data will be presented as numbers and percentages with comparisons in proportions made at 6 weeks and 6 months	Intermediate and end point
Partner involvement during pregnancy, at 6 weeks and at 6 months	Maternal/paternal self-report	Partner involvement in child care	Information on partner involvement in child care will be collected by trained interviewers using a semi-structured questionnaire	Categorical data will be presented as numbers and percentages with comparisons in proportions made at 6 weeks and 6 months	Baseline, intermediate and end point

*SD* standard deviation, *WHO* World Health Organization

### Formative and process data

Study procedures were informed by formative qualitative research conducted with pregnant women attending antenatal care at CHBH prior to the trial inception. We have written standard operating procedures (SOPs) to support the research team to deliver the study procedures and intervention according to the protocol. Participant adherence will be assessed by counts (e.g., visit attendance) and intervention delivery will be monitored by observing contacts between the sonographer (who will conduct the US and deliver the ECD messages) and participants (intervention and control) to record the nature of interactions. We will monitor intervention dose and experience by recording the duration of the ultrasound visit (with and without delivery of the intervention) and by gathering participant and partner views on the ultrasound experience. Ongoing consultation with a key stakeholder network will inform intervention procedures and assist with contextualising the intervention and addressing any challenges that may arise during trial implementation.

Due to the well-documented challenges with engaging men in research and health care in South Africa [54], we will adopt pro-active recruitment strategies, which will include telephone calls to partners after participant enrolment and follow-up calls closer to the visit to encourage attendance. We have also included Saturday sessions for those partners who are unable to attend during the week due to work commitments. Research staff will make regular contact with participants in-between study visits to maintain the accuracy of records and to promote retention of participants in the study. We will follow-up participants who default and those who withdraw to gain an understanding of why they dropped out during the course of the trial.

### Data analysis

All available data at baseline will be analysed descriptively for each intervention group and in total. Categorical data will be summarised by numbers and percentages. Continuous data will be summarised by the mean, SD and range if data are normal and by the median, IQR and range if data are skewed. Minimum and maximum values will also be presented for continuous data. Differences regarding baseline variables will be checked for clinical relevance between the three treatment groups.

A CONSORT flow diagram will be used to summarise the number of participants who were: assessed for eligibility at screening, eligible at screening, eligible and randomised, lost to or discontinued the intervention and included in the primary outcome analysis. If possible, respondents and non-respondents will be compared.

The analysis of the primary outcome will be performed on the intention-to-treat population with no imputation of missing data. In the primary analysis, child development scores will be compared to the control group. If outcome data are normally distributed, we will fit linear regression models. If not normally distributed, outcome data will be categorised and logistic regression methods will be used to assess differences between intervention and control groups. Tests will be two-sided with a significance level of 0.05, and 95% confidence Intervals will also be reported. In case of relevant differences in baseline characteristics, additional adjustment(s) for these factors will be performed within the models to address potential confounding.

A stratified analysis will be done for three pair-wise comparisons—that is, Arm 1 vs. control, Arm 2 vs. control and Arm 1 vs. Arm 2 —according to the following subgroups: partner vs. no partner accompaniment and parity.

STATA, Version 15 (StataCorp, College Station, TX, USA) will be used for all analyses. Missing data and data from those who withdraw from the study will not be interpreted.

## Discussion

While ultrasound examination has long been regarded as having potential to be transformative and to improve parental behaviour [14], as well as commitment to the growing child [12], this hypothesis has only been tested in a few studies in high-income countries, fewer of which were randomised control trials [55].

There is clear evidence from high-income countries that the first time a prospective mother sees an image of their fetus during US is an emotionally enjoyable experience, which parents report intensifies their attachment to the coming baby. This is true also of prospective fathers [22, 56], and is reported as being an important bonding experience for the family [57]. A number of studies have examined the impact of ultrasound examination on maternal attachment [24, 58, 59]. However, women’s experiences of US are dependent on the context and the behaviour of the sonographer, with some women reporting feeling objectified and poorly informed during the experience [60].

Research on the emotional and relationship effects of early US are important because maternal (and presumably paternal) representations of the coming baby are significant predictors of parent–infant interactions which, in turn, affect healthy infant development [61, 62]. A recent review of interventions indicated that awareness programmes, most commonly involving pregnancy US, were amongst the most promising interventions to increase parent–fetal attachment [55]. There is agreement that studies are needed which follow up parental–child behaviour and healthy child development postnatally, include fathers and examine the construct in a wider diversity of settings, including in low and middle-income countries. Testing the added benefits of pregnancy US for child development is the gap that this proposed randomised control trial in South Africa seeks to address.

Should this trial demonstrate feasibility and improvement in the specified intermediate and outcome measures, the next step would be a pragmatic trial situated in the health-care system, with longer-term outcomes and cost-effectiveness estimations.

### Dissemination policy

The authors will share the study results with the trial stakeholder network, including the study participants (where requested), the South African Ministry of Health and relevant clinical, academic and research partners. The authors will also publish the results after study completion. Authorship will include the co-investigators as well as any postgraduate students who may contribute substantially to analysis and writing of a particular paper. Professional writers will not be used.

### Trial status

The trial protocol, version number 2.0, was approved on 16 January 2019 and is ongoing. The recruitment began on 1 March 2019 and was completed on 30 September 2019. Trial procedures are expected to be complete by the end of July 2020.

## Data Availability

The questionnaires and coding sheets used in the study, as well as anonymised data, will be included in or as supplementary files to articles published, as required. Trial results will be disseminated to participants and relevant health-care providers via a stakeholder committee.

## Abbreviations

ANC: Antenatal clinic
Bayley: III Bayley Scales of Infant and Toddler Development, Third Edition
CHBH: Chris Hani-Baragwanath Hospital
DPHRU: Developmental Pathways to Health Research Unit
ECD: Early childhood development
FMU: Foetal Medicine Unit
HIC: High-income country
NDoH: National Department of Health
SA: South Africa
US: Ultrasound scan
WHO: World Health Organization
